# Desert truffle mycorrhizosphere harbors organic acid releasing plant growth–promoting rhizobacteria, essentially during the truffle fruiting season

**DOI:** 10.1007/s00572-021-01067-w

**Published:** 2022-01-18

**Authors:** Francisco Arenas, Álvaro López-García, Luis Miguel Berná, Asunción Morte, Alfonso Navarro-Ródenas

**Affiliations:** 1 Dpto. Biología Vegetal (Botánica), Facultad de Biología, Universidad de Murcia, CEIR “Campus Mare Nostrum”, Campus de Espinardo, 30100 Murcia, Spain; 2 Department of Soil Microbiology and Symbiotic Systems, Estación Experimental del Zaidín-CSIC, Calle Prof. Albareda, 18008 Granada, Spain; 3 Department of Animal Biology, Plant Biology and Ecology, Universidad de Jaén, Jaén, Spain; 4 Instituto Interuniversitario de Investigación del Sistema Tierra en Andalucía (IISTA), Av. del Mediterráneo, 18006 Granada, S/N Spain

**Keywords:** Desert truffle, *Terfezia*, *Helianthemum*, PGPR, P-solubilizing, ACC-deaminase, Ectendomycorrhiza

## Abstract

Desert truffle is becoming a new crop in semiarid areas. Climatic parameters and the presence of microorganisms influence the host plant physiology and alter desert truffle production. Desert truffle plants present a typical summer deciduous plant phenology divided into four stages: summer dormancy, autumn bud break, winter photosynthetic activity, and spring fruiting. We hypothesize that the bacterial community associated with desert truffle plants will show a seasonal trend linked to their plant growth–promoting rhizobacteria (PGPR) traits. This information will provide us with a better understanding about its potential role in this symbiosis and possible management implementations. Bacteria were isolated from root-adhering soil at the four described seasons. A total of 417 isolated bacteria were phenotypically and biochemically characterized and gathered by molecular analysis into 68 operational taxonomic units (OTUs). They were further characterized for PGPR traits such as indole acetic acid production, siderophore production, calcium phosphate solubilization, and ACCD (1-amino-cyclopropane-1-carboxilatedeaminase) activity. These PGPR traits were used to infer functional PGPR diversity and cultivable bacterial OTU composition at different phenological moments. The different seasons induced shifts in the OTU composition linked to their PGPR traits. Summer was the phenological stage with the lowest microbial diversity and PGPR functions, whereas spring was the most active one. Among the PGPR traits analyzed, P-solubilizing rhizobacteria were harbored in the mycorrhizosphere during desert truffle fruiting in spring.

**Supplementary information:**

The online version contains supplementary material available at 10.1007/s00572-021-01067-w.

## Introduction

Desert truffle cultivation is becoming a new agricultural activity in semiarid areas of the Iberian Peninsula because of the low water input required for its cultivation (Morte et al. 2009). Nowadays, *Terfezia claveryi* Chatin cultivation with *Helianthemum* spp. as host plants is a reality, where *T. claveryi* is one of the few mycorrhizal fungal species that is being cultivated (Honrubia et al. 2001; Morte et al. 2008). Since 1999, many plantations of different sizes, with *T. claveryi* and several perennial *Helianthemum* shrubs, have been established, and new strategies have been developed to increase the scale of mycorrhizal plantlet production (Morte et al. 2012; Morte and Andrino 2014; Navarro-Ródenas et al. 2016). Desert truffle plantations usually start to produce 2 to 3 years after planting. Carpophores are yearly produced and production is abundant if autumn and spring rainfalls occur (Andrino et al. 2019). A proper irrigation or precipitation scheduling is one of the most important factors for maintaining successful cultivation (Honrubia et al. 2014). Andrino and colleagues (2019) showed that desert truffle production strongly correlates with autumn rainfall (Morte and Andrino 2014) and to a lesser extent with spring rainfalls and vapor pressure deficit (Andrino et al. 2019; Marqués-Gálvez et al. 2020a). However, other agroclimatic parameters such as temperature, relative humidity, soil water potential, and soil nutrients or the presence of other microorganisms have been shown to influence the desert truffle plant physiology along the plant phenology and hence have the potential to affect desert truffle production (Morte et al. 2010; Navarro-Ródenas et al. 2011, 2012, 2013, 2016; Andrino et al. 2019).

The host plant *Helianthemum almeriense* Pau presents a typical summer deciduous plant phenology (Morte et al. 2010; Flexas et al. 2014; Marqués-Gálvez et al. 2020a), with a conservative water use strategy, mainly based on the avoidance of drought stress by reducing the stomatal conductance in late spring (May) and finally on losing its leaves during summer (Morte et al. 2010; Marqués-Gálvez et al. 2020a). In early autumn, when the temperature decreases and with the first rainfalls after summer, buds break and new fine roots are produced. The rainfall during autumn seems to be crucial for desert truffle fruiting during the next spring. In fact, Bordallo (2007) observed truffle primordia in the rhizosphere of *Helianthemum* sp. in autumn. After bud break, photosynthesis begins to increase, reaching its maximum during January–February. This period of maximum photosynthesis occurs just before plant blooming and desert truffle fruiting (March–May). Thus, we can divide desert truffle plant phenology into four stages: (i) summer dormancy (June–August); (ii) bud break (September–October); (iii) maximal photosynthetic activity (January–February); and (iv) plant blooming and desert truffle fruiting season (March–May). This yearly cycle was shown to be important and necessary for plant fitness and desert truffle production (Morte et al. 2012; Honrubia et al. 2014).

Recently, it has been seen that mycorrhizal roots, mycorrhizosphere soil, and peridium of desert truffles are enriched in plant growth-promoting rhizobacteria (PGPR) and mycorrhizal helper bacteria (MHB), and the direct effects of some of them on increasing survival rates and mycorrhization of *H. almeriense* plants have been highlighted in nursery conditions (Navarro-Ródenas et al. 2016). In order to manage ecofriendly crops, such as desert truffles, the application of biofertilizers based on PGPR is an asset (Basu et al. 2021). However, there is little information available about the PGPR activities that could be important during each phenological stage. Because of the marked seasonality of the desert truffle ecosystem under study, we hypothesize that the PGPR traits of the bacterial community associated with the mycorrhizosphere of desert truffle plants (*Helianthemum*-*Terfezia* symbiosis) will shift during the different plant phenology stages. The PGPR mechanisms include direct or indirect activities such as phosphate solubilization, production of PGPR molecules (auxins), reduction of ethylene precursor levels (ACC) in plants, and secretion of iron chelates (Lugtenberg and Kamilova 2009; Azcón 2014; Jha and Saraf 2015), which all have an impact on plant nutrition and physiology, and which may elicit antagonistic effects against phytopathogenic microorganisms (Prasad et al. 2015). Molecular methods, based on 16S rRNA amplicon data, have relied in the use of databases for the ecological predictions of community functional traits (Langille et al. 2013). However, there are serious limitations to link sequencing data with microbial functions because a low percentage of ecologically relevant strain-specific genes has been identified (Goberna and Verdú 2016; Fernández et al. 2019). Culture-dependent and molecular sequencing methods have already been used to describe the bacterial diversity associated with different appreciated truffle species of *Tuber*, but their functionality remains largely unexplored (Barbieri et al. 2016). While many studies have focused on the microbial community composition, only a few of them have tested different PGPR activities of bacteria isolated from truffle ecosystems (Adeleke and Dames 2014; Barbieri et al. 2016; Chen et al. 2019). To trace the functional dynamics of the potential PGPR, we used cultivation-based methods that rely on the isolation, identification, and trait characterization of the PGPR communities across seasons (Cadotte et al. 2011; Chauhan et al. 2015). Specifically, we aimed to understand the functional dynamics of the cultivable PGPR associated with *T. claveryi*, across the different stages of plant phenology and, hence, we will delve into the functioning of desert truffle ecosystem. In the light of the increasing interest in studying the role of microbiomes in providing ecosystem services (i.e., as biofertilization and biocontrol uses) (Kumar et al. 2020), this knowledge could be used to implement co-inoculations of *T. claveryi* with beneficial bacteria to increase ascocarp yields and enhance a better management of desert truffle plantations.

## Materials and methods

### Sampling collection

*H. almeriense* × *T. claveryi* rhizosphere soil and root samples were carefully collected from a productive man-planted plot in Zarzadilla de Totana, Murcia (Spain), at different *H. almeriense* phenological stages during autumn (October 2014), winter (January 2015), spring (April 2015), and summer (July 2015) season. Four soil samples in autumn and three samples in winter, spring, and summer from approximately 20 cm from the plant and separated by a minimum distance of 5 m were collected. The first 5-cm soil surface was carefully removed and a cylinder of soil of approximately 10 cm of diameter and 15 cm of depth bearing roots was sampled. All samples were kept in sterile plastic bags and transported at 4 °C. In the lab, 0.5 g of *H. almeriense* fine roots, randomly selected from total root system, was carefully taken to avoid losing adhered soil and transferred into 250-mL Erlenmeyer flasks containing 100 mL of sterile Ringer one-fourth solution and one drop of Tween-20. Flasks were shaken at 150 rpm for 60 min. Serial dilutions were prepared and 0.1-mL aliquots (10^−3^ to 10^−6^) were spread on Nutrient Agar (NA) solid medium plates. The plates were incubated for 72 h at 30 °C. Colonies appearing on the medium were counted at 24, 48, and 72 h in order to calculate colony-forming units per gram of sample (CFU g^−1^). From those dilution plates ranging from 30 to 300 cfu/plate, 34–35 colonies/samples were randomly isolated on plates with the same medium, with a total of 104 colonies per plant phenology stage (season). The isolated strains were routinely subcultivated on NA plates and long-term stored in Nutrient Broth (NB) amended with 25% glycerol at −80 °C.

### Colony characterization

Isolated colonies were defined by color, shape, edge, and texture (waxy, mucilaginous, pulverulent, or aqueous). All strains were characterized by Gram staining and phase contrast microscopy (size, shape, motility, and spore) (Bartholomew and Mittwer 1952). Biochemically, they were defined by catalase, oxidase, starch hydrolysis, and lipid hydrolysis. In addition, the fluorescence of the colonies was qualitatively checked under UV light on agar plates.

Bacterial colonies were sorted into phenotypical groups based on the aforementioned phenotypic characteristics. Then one out of five colonies from each phenotypic group was PCR-amplified using the 16S rDNA primers, 27F (5′AGAGTTTGATCMTGGCTCAG3′), and 1492R (5′TACGGYTACCTTGTTACGACTT3′) (Weisburg et al. 1991). PCR was performed using recombinant *Taq* DNA polymerase (Invitrogen) according to the manufacturer’s instructions. Colonies approximately 1 mm in diameter were picked up with a sterilized toothpick and directly transferred to the PCR tubes as DNA templates. PCR additives and thermal cycle program followed Navarro-Ródenas et al. (2016). The PCR products were sequenced by the dideoxy sequencing method (Sanger et al. 1977) using the ABI Prism 310 (Applied Biosystems, Foster City, CA, U.S.A.) at the Molecular Biology Service of the University of Murcia. The nucleotide sequences of the 16S rDNA were aligned through MUSCLE algorithm using the software MEGA version 7.0 (Kumar et al. 2016) and sequences with similarity higher than 97% were clustered into the same Operational Taxonomic Units (OTUs) using MOTHUR software (Schloss et al. 2009). Then, each OTU was subjected to BLAST analysis (Altschul et al. 1990) against the NCBI database (http://blast.ncbi.nlm.nih.gov/Blast.cgi) in order to assign it a tentative taxonomical category.

Finally, an OTU abundance table was built for subsequent statistical analyses by extrapolating the number of colonies with the same phenotype to the number of sequenced colonies for each season (Table S1).

### Screening for PGPR activities

Cultivable bacterial colonies from the OTUs obtained were further characterized qualitatively for plant growth-promoting traits: indole acetic acid production (IAA), siderophore production, phosphate solubilization, and 1-amino-cyclopropane-1-carboxilate deaminase (ACCD) activity.

IAA production was measured by a colorimetric method (Gordon and Weber 1951). For this, the isolates were cultivated in NB medium supplemented with 3 g L^−1^ of tryptophan (Ahmad et al. 2005; Leveau and Lindow 2005) at 30 °C for 2 days in a shaking incubator, at 100 rpm. Bacterial cells were removed from the culture broth by centrifugation (1.5 mL of bacterial suspension). Supernatants were vigorously mixed in a 1:4 ratio with Salkowski’s reagent (Rahman et al. 2010; Goswami et al. 2014) and incubated in the dark for 30 min at 25 °C. Presence of IAA produced was detected through a change in color to pink.

Estimation of siderophore production was determined using an Fe-deficient mineral salt medium (MM9) (Radzki et al. 2013). The strains were inoculated in MM9 and incubated in a shaking incubator at 30 °C for 2 days at 100 rpm. The cell-free culture supernatants were assayed for detection of siderophores secreted by bacteria using a commercially assay kit, SideroTec Assay™ (http://www.emergenbio.com/), which can be used for detection of a wider range of iron-binding compounds (Odoni et al. 2017; Ankley et al. 2020). A total of 100 µL of supernatants was mixed with 100 µL of the pre-mixed R1 reagent/R2 and incubated for 10 min at room temperature following the protocol provided by the kit. Siderophore presence was detected with a change in color to purple or pink.

Phosphate solubilization by PGPR strains was quantified using solidified medium containing tricalcium phosphate as the only source of phosphorus in modified National Botanical Research Institute’s phosphate growth medium (NBRIP) (Nautiyal 1999) supplemented with bromophenol blue (Chen et al. 2006; Pande et al. 2017). The strains were incubated at 28 °C for 5 days. Bacterial strains developing clear zones around their colonies (halo presence) on agar plates were identified as P-solubilizing.

An indirect assay was carried out for screening of ACCD activity by bacterial isolates, based on the bacterial ability to use ACC (the ethylene precursor metabolite in plants) as a nitrogen source in a similar way as was described by Ambrosini and Passaglia (2017). Bacterial strains were grown in 5 mL NB medium for 24 h at 100 rpm at 28 °C. Bacterial culture was centrifuged at 8000 rpm for 5 min and the supernatant was removed. The cell pellet obtained was washed with sterile Ringer one-fourth solution twice and resuspended in 1 mL of Ringer one-fourth solution. Then, bacterial suspension was spot-inoculated on agar plates containing DF salts supplemented with 6 mM of ACC and without ACC (negative control), or supplemented with 0.4 g L^−1^ of (NH_4_)SO_4_ as positive control (Penrose and Glick 2003; Martínez et al. 2018). The plates were incubated for 3–4 days at 28 °C and colony growth was evaluated. The growth of isolates on ACC-supplemented plates was compared with positive and negative control plates for ACCD strain characterization.

### Phenological characterization of desert truffle mycorrhizal plants

At the same time of soil sampling, plant phenological status (bud flushing, blooming, flowering, and leaves senescence) in the plantation was described. Photosynthesis and stomatal conductance were estimated using a portable photosynthesis system (LI-6400, Li-Cor, Inc., Lincoln, NE, USA) equipped with an integrated fluorescence chamber head (Li-6400–40; Li-Cor). Shoot water potential (Ψ_shoot_) was measured in 5-cm-long plant apex cut and immediately placed in a pressure chamber (Soil Moisture Equipment Co; Santa Barbara, CA, USA) according to Scholander et al. (1965). All these quantitative parameters were recorded in six mycorrhizal plants per season, as previously described in Morte et al. (2010), Navarro-Ródenas et al. (2013), and Marqués-Gálvez et al. (2020a).

Mycorrhizal colonization was assessed in the roots of six plants by season. Frozen fine roots were randomly selected, stained, and observed under a light microscope for mycorrhizal percentage calculation as previously described by Gutiérrez et al. (2003).

### Statistical analysis

The OTU abundance table (Table S1) was Hellinger transformed prior to multivariate analyses. The effect of seasonality was tested by permutational multivariate analysis of variance (PERMANOVA, (McArdle and Anderson 2001); *adonis* function, *vegan* R package). Since the OTU abundance matrix was previously Hellinger transformed, using Euclidean distance as measure of dissimilarity is equivalent to using a Hellinger-based distance (Legendre and Gallagher 2001). To discard the differences in multivariate dispersion across seasons was driving the patterns found in PERMANOVA; differences in multivariate dispersion across seasons were checked (*betadisper* function, *vegan* R package). A Non-metric multidimensional scaling (NMDS) ordination was used to visualize the found patterns using Euclidean distance (Hellinger-based) as measure of dissimilarity.

An RLQ analysis was carried out (Dray and Legendre 2008) to study if seasonality was driving the PGPR activities at community level. RLQ tests the link between three matrices: a species/OTU abundance (abundance of OTUs in columns, in each sample, in rows), a trait (OTUs in rows × traits, presence/absence of PGPR activities, in columns), and an environmental matrix (season, in column × sample, in rows). This analysis considers the averaged PGPR activity at community level to calculate the statistical significance of the link between environment (in this case season) and species traits. An abundance table including only those OTUs with any PGPR activity was generated and the link between the mentioned matrices was tested using the *randtest.rlq* procedure (*ade4* R package) using 9999 permutations. The effect was tested using the permutation model #6, which is a combination of models #2 (permute values of sites) and #4 (permute values of species) and does not have an inflated type I error (Dray and Legendre 2008; Braak et al. 2012).

To test for the particular relationship between season and PGPR activities in bacterial communities, community-weighted means (CWMs) of PGPR activities were calculated using *funtcomp* function (*FD* R package) and the OTU abundance table and the presence (1) or absence (0) of each PGPR activity in the OTUs. Differences in PGPR CWMs across seasons were tested by analysis of variance (ANOVA); when they were significant, multiple comparisons between means were arranged by means of *t*-test corrected for multiple comparisons (Bonferroni) as post hoc. The statistical significance threshold was fixed at *p* ≤ 0.05. We tested normality with Shapiro–Wilk test, and homoscedasticity with Levene’s test. When assumptions were not met, the non-parametric Kruskal–Wallis test was applied. If significant, a Dunn’s test corrected by Bonferroni post hoc was performed.

## Results

**Fig. 1:**
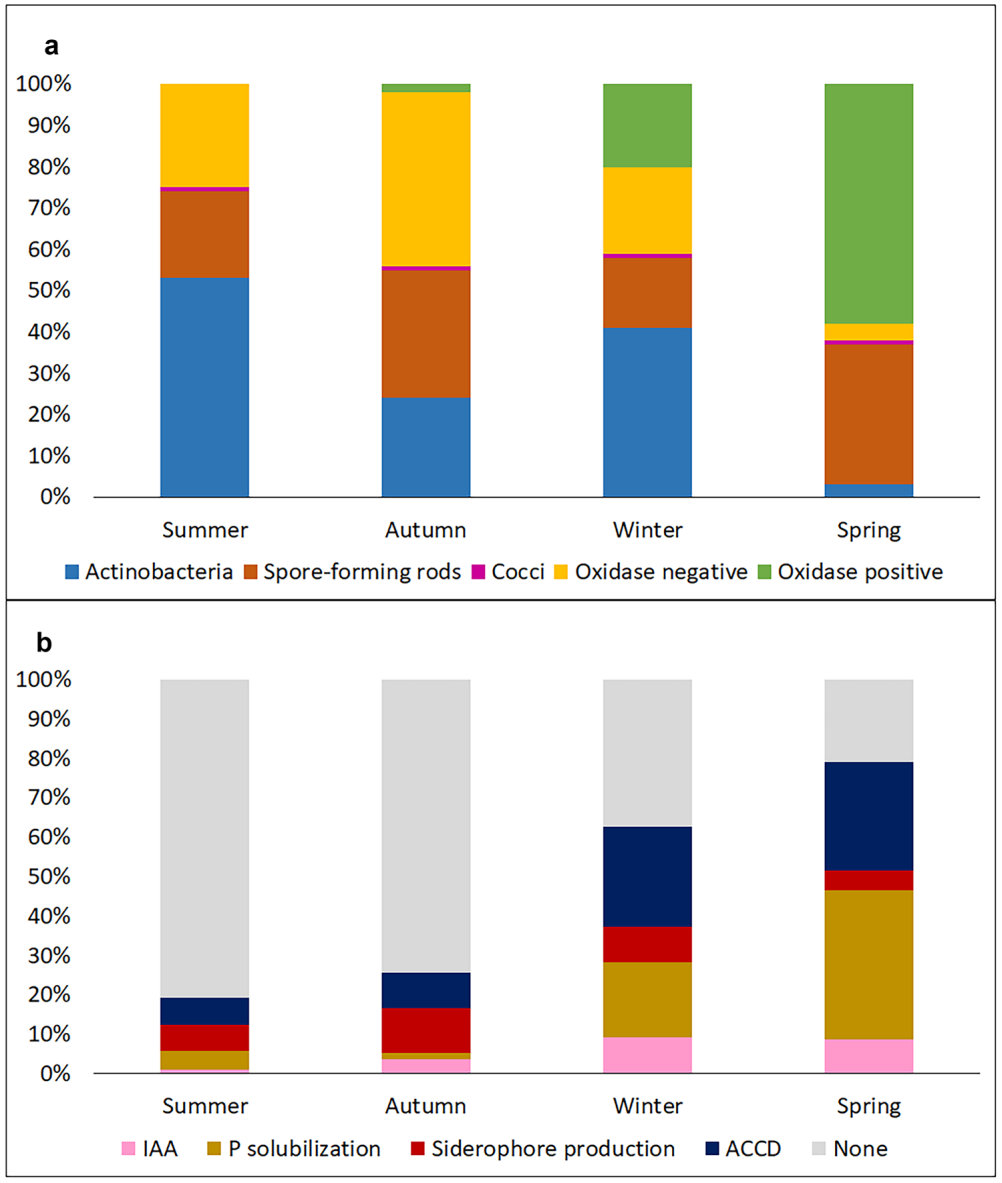
Mycorrhizosphere bacteria composition during seasons **a** based on microscopy and biochemical phenotype characterization of 417 isolated strains and **b** based on plant growth-promoting traits in the 68 different OTUs for auxin production (IAA), phosphate solubilization (P solubilization), siderophore production, ACC deaminase production (ACCD), or PGRP trait not detected (none)

In the four sampled seasons, 417 cultivable colonies were obtained from *H. almeriense* × *T. claveryi* mycorrhizosphere by non-selective media. Among the isolates, a slightly higher proportion of Gram-positive bacteria (57%) than Gram-negative bacteria (43%) was observed (Fig. 1a, Table S2). However, the relative proportion between Gram-positive and negative bacteria showed a seasonal trend. In summer, the highest percentage of Gram-positive bacteria (75%) was observed, represented mainly by filamentous bacteria (Fig. 1a, Table S2). The percentage of Gram-positive bacteria decreased during autumn (56%) and winter (59%), reaching the lowest values in spring, when Gram-positive bacteria represent 38% of the total, mainly represented by spore-forming rods (Fig. 1a, Table S2). Among Gram-negative bacteria, both oxidase-negative and positive bacteria were found and their relative abundance switched from summer (25%) to spring (62%), and they were oxidase-negative dominant in summer (25%) and autumn (42%) and oxidase-positive dominant in spring (58%) (Fig. 1a, Table S2).

Based on the phenotypic characterization, the 417 colonies were sorted in 72 phenotypical groups and some of them were merged into the same OTU by the molecular analysis of the 16S rDNA partial sequence gene (Table S1), resulting in 68 different OTUs. Out of 68 PGPR trait-characterized OTUs, 28 (41%) did not exhibit any of the PGPR activities assayed in this study, 40 (59%) exhibited at least one activity, 21 (31%) exhibited at least two different activities, and only 4 (6%) exhibited three activities (Fig. 1b, Table S3). Of the isolated strains, 11 (16%) were found to produce IAA, 16 (24%) produced phosphate solubilization, 17 (25%) exhibited the ability to release siderophores, and 21 (31%) showed ACCD production (Fig. 1b, Table S3). These OTUs belonged to the following 15 genera with different percentage of abundance in terms of isolated colonies: *Streptomyces* (19.4%), *Pseudomonas* (18.9%), *Bacillus* (18.2%), *Sinorhizobium* (13.4%), *Paenibacillus* (7.4%), *Actinomyces* (7.2%), *Staphylococcus* (6.0%), *Arthrobacter* (3.4%), *Variovorax* (3.4%), *Acinetobacter* (1.0%), *Bradyrhizobium* (0.7%), *Brevibacillus* (0.2%), *Chitinophaga* (0.2%), *Micrococcus* (0.2%), and *Stenotrophomonas* (0.2%) (Table S4).

**Fig. 2:**
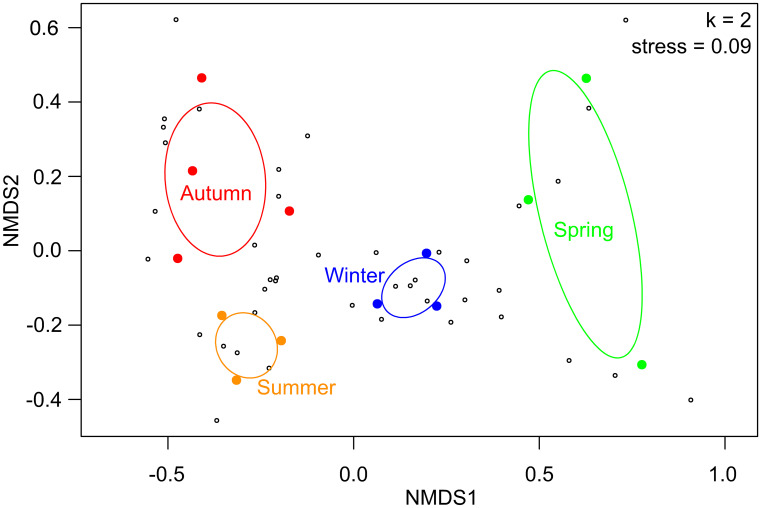
Non-metric multidimensional scaling ordination of isolated bacterial communities associated with desert truffle plants in different seasons. Filled circles denote samples, open circles denote bacterial OTUs. Ellipses denote 95% confidence intervals. Permanova results regard season showed significant differences (*F* = 2.7061, *p* = 0.001, *R*^2^ = 0.474)

**Fig. 3:**
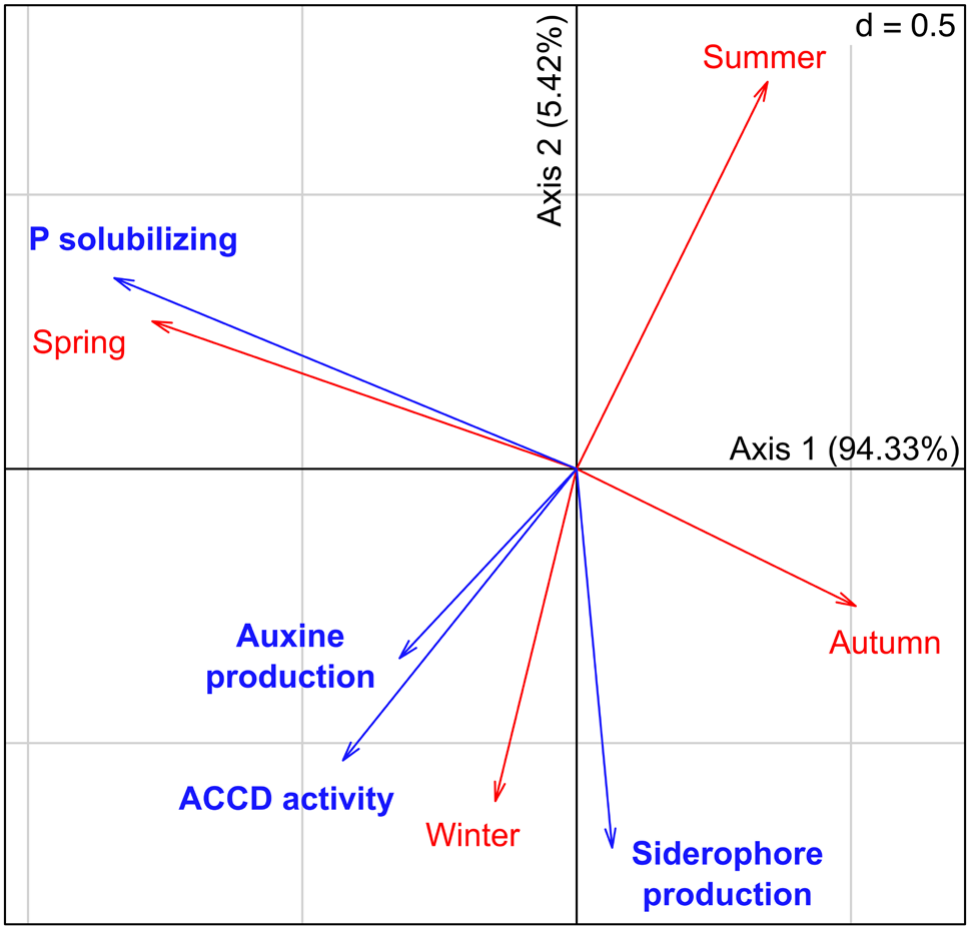
RLQ joined ordination showing the relationship between PGPR traits and seasons from bacterial isolates. Direction and length of vectors indicate correlation with other variables and contribution to the ordination, respectively (model #2, *p* = 0.0045; model #4, *p* = 0.0004)

**Fig. 4:**
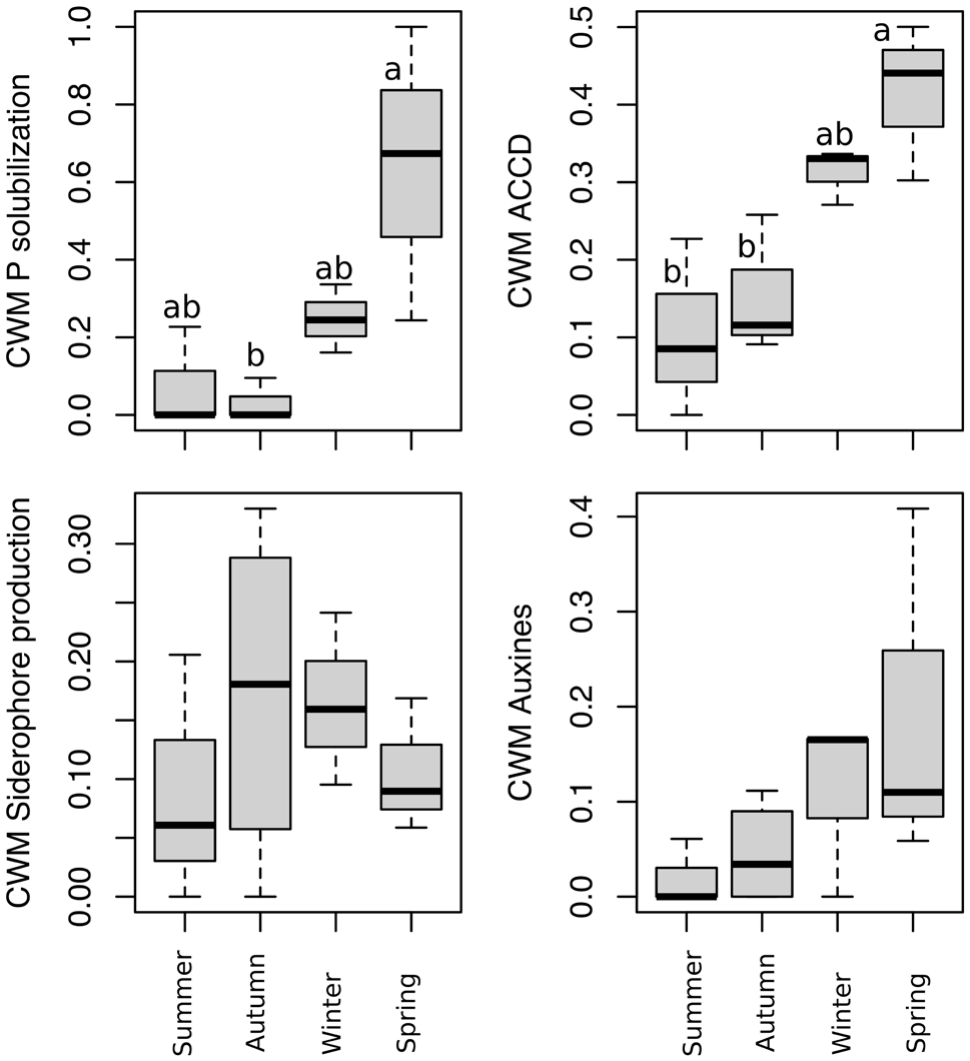
Community weighted means (CWMs) analysis of the PGPR activities in bacterial colonies across seasons. Different letters indicate significant differences between groups (*p* < 0.05)

The composition of the cultivable bacterial community obtained varied across plant phenological stages (i.e., seasons) as shown in the PERMANOVA (*F* = 2.706, *p* = 0.001, *R*^2^ = 0.474) (Fig. 2; Table S5.1). This result was not an artifact caused by a differential beta dispersion among treatments since the multivariate dispersion was constant between seasons (*F* = 1.095, *p* = 0.400; Fig. 2). An overall significant relationship between the seasons and the bacterial community PGPR traits was found (RLQ analysis: model #2, *p* = 0.0045; model #4, *p* = 0.0004, Table S5.2, Fig. 3), which means that the change in the OTU composition implied a change in the functionality of the bacterial communities across seasons. The subsequent lineal models applied to CWMs of PGPR activities revealed a significant effect of season on P-solubilizing (*df* = 3; Kruskal–Wallis *χ*^2^ = 9.157; *p* = 0.027) and ACCD activity (*df* = 3, 9; *F* = 8.892; *p* = 0.005) (Fig. 4). According to this analysis, there were two periods of the year when the CWMs of both P-solubilizing and ACCD activity were different. Both PGPR activities showed low values in autumn and high values in spring. CWMs of IAA–producing and siderophore production were statistically constant across seasons (Fig. 4).

In summer, the percentage of *Terfezia* mycorrhizal roots dropped to a minimum (Table 1). Almost no plant and/or fungal activity was observed. The amount of CFU g^−1^ of root recorded was the lowest (1.61 ± 0.6 × 10^6^) (Table S2). Eight out of 68 OTUs were exclusively isolated in summer (Table S1). According to the CWMs analyses, summer was the season with the lowest PGPR activities obtained (Fig. 4). Indeed, most bacterial isolates did not show any of the studied activities (Tables S1, S3).

**Table 1:** Annual phenological characterization of desert truffle mycorrhizal plants during the experimental year in a plantation

Season	Plant status	Gas exchange parameters	Mycorrhization
Autumn	Bud break	*A*: 2.89 µmol·m^−2^·s^−1^ *gs*: 0.14 mmol·m^−2^·s^−1^ ψ_shoot_: − 1.69 MPa	50–80% intracellular
Winter	Vigorous vegetative growth Flower buds	*A*: 5.41 µmol·m^−2^·s^−1^ *gs*: 0.10 mmol·m^−2^·s^−1^ ψ_shoot_: − 1.46 MPa	13–48% intercellular
Spring	Blooming Desert truffle production	*A*: 1.56 µmol·m^−2^·s^−1^ *gs*: 0.03 mmol·m^−2^·s^−1^ ψ_shoot_: − 1.77 MPa	50–80% intracellular
Summer	Leaf senescence	*A*: ND *gs*: ND ψ_shoot_: ND	0–15% intracellular

*ND* not detectable

During autumn, the percentage of *Terfezia* mycorrhization rose quickly and the photosynthetic rate began to increase (Table 1). During this period, the amount of CFU g^−1^ of root recorded was the highest (4.52 ± 3.0 × 10^7^) (Table S2). Fifteen out 68 OTUs were exclusively isolated in autumn (Table S1). The CWMs analyses revealed that autumn had still significantly lower values than spring season for phosphate solubilizing and ACC deaminase–producing bacteria (Fig. 4).

In winter, *H. almeriense* also continued to develop new shoots and leaves and showed the highest gas exchange parameters as photosynthesis and stomatal conductance along the annual cycle (Table 1). The percentage of *Terfezia* mycorrhization was not as high as in autumn (13–48%) and intercellular colonization was dominant (Table 1). In this period, the CFU g^−1^ of root was around 3.28 ± 1.3 × 10^6^ (Table S2). Sixteen out of 68 OTUs were exclusively isolated in winter (Table S1).

The most important period is spring, when desert truffles fructify. The experimental plantation yielded a desert truffle production of 277.6 kg/ha that was harvested some days after sampling. Fungal fructifications were observed at the same time of blooming and the photosynthetic parameters in *H. almeriense* plants dropped from winter on Table 1. CFU g^−1^ of root was 2.03 ± 1.5 × 10^6^ (Table S2). Ten of 68 OTUs were exclusively isolated in spring (Table S1). According to the RLQ analysis (Fig. 3; Table S5.2) and CWM tests, there were significant variations with ACCD producer bacteria and phosphate solubilizer bacteria (Fig. 4). The highest values of CWMs of these PGPR activities were found in this season (Fig. 4), mainly represented by *Pseudomonas* and *Paenibacillus* spp. (Table S4).

## Discussion

Desert truffle plants confirmed a very clear phenology along the year with different milestones in autumn, winter, spring, and summer according to the results of this work (Table 1) and as previously reported by Andrino et al. (2019) and Marqués-Gálvez et al. (2020a, b). Although Andrino et al. (2019) showed that all plant and fungal changes and developments, from summer to spring, should be crucial for proper fruiting and crop yield, they also confirmed the experience of some gatherers and farmers, related to the importance of the two key periods: autumn and spring.

According to RLQ and CWM analyses, the abundance of bacteria with certain PGPR traits was significantly enriched in spring compared to autumn (Fig. 3). Here, we showed that not every PGPR trait varied in the same way across seasons. Abundance of siderophore producer and auxin releaser bacteria was maintained almost constantly along the year. But ACCD and P solubilization bacteria abundance fluctuated with the two key periods of this crop, being low in autumn and high in spring.

The bud breaking observed in autumn (Table 1) could be related with low ACCD bacteria abundance. Autumns are usually rainy in the Mediterranean area, but scarce rainfall during autumn has been correlated with low desert truffle production the next spring (Andrino et al. 2019). Among the effects observed on desert truffle plants, during dry autumns is the delay in bud breaking. It has been reported that bud break is enhanced by potassium cyanide (KCN), a co-product of the ethylene production from ACC (Mizutani et al. 1994). Furthermore, Mizutani et al. (1994) and Tohbe et al. (1998) reported that exogenous ACC application promoted bud break of grape buds. Desert truffle plants could disfavor ACCD bacteria since their presence could result in a sink for ACC and consequently reduce its level within the plant (Saraf et al. 2010), if low ACC level could inhibit desert truffle (mycorrhizal plant) bud breaking in autumn.

High spring ACCD bacteria abundance correlated with the absence of leaf senescence (Table 1). It has been reported that leaf senescence, the end of both the plant flowering and desert truffle fruiting seasons, is initiated by the increase in vapor pressure deficit (VPD) during late spring (Andrino et al. 2019; Marqués-Gálvez et al. 2020a). Ethylene is one of the most important hormones in triggering the leaf senescence process. The ACC content only increases in senescing leaves, in parallel with the ethylene production (Hunter et al. 1999), and it inhibits flowering in some species (Achard et al. 2007). If the phenological switch reported in desert truffle plants (Marqués-Gálvez et al. 2020a) is mediated by ethylene, the presence of ACCD producer bacteria could reduce the effect of the VPD on plant phenological switch.

P-solubilizing bacteria were clearly related with a season and consequently with the plant and fungal activity at that time. Phosphorus is an essential macronutrient for plants, usually limiting photosynthesis in terrestrial ecosystems (Reich et al. 2009). But in this work, the high presence of P-solubilizing bacteria was not related to high photosynthesis values in spring (Table 1). Indeed, the photosynthesis in spring, according to Marqués-Gálvez et al. (2020a, b), was already close to its minimum due to the limitation by VPD and/or drought. In spring, the fungal partner, however, produces its fruiting bodies (Table 1) and high metabolic activity should occur belowground, justifying a higher demand of nutrients. We should remember that phosphate solubilization is induced through the release of organic acids, necessary to lower the pH (Adnan et al. 2017) in alkaline soils. Together with phosphorus, other elements such as potassium, sulfur, iron, or manganese are also released (Etesami and Adl 2020). Although *T. claveryi* is naturally restricted to calcareous alkaline soil (Zambonelli et al. 2014), Arenas et al. (2018) showed that *T. claveryi* grows better at pH 5, in in vitro conditions. In addition, Navarro-Ródenas et al. (2016) isolated a strain of *Pseudomonas mandelii* #29 from the peridium of *T. claveryi* truffles. This strain exhibited a high phosphate-solubilizing capacity through the release of organic acids. It was considered as a mycorrhiza helper bacterium (MHB) since it promoted root mycorrhizal colonization but not plant growth (Navarro-Ródenas et al. 2016; Espinosa-Nicolás 2017; Martínez-Ballesteros 2018).

According to these results, it seems reasonable that truffle plant mycorrhizosphere could select for bacterial communities enriched in particular functions to foster their phenological advancement. Indeed, the ability of plants to select their accompanying microbiome has already been observed (Bever et al. 2009; Kiers et al. 2011). Moreover, it is also plausible that the desert truffle mycorrhizosphere actively selects those bacteria which are able to change the microenvironmental pH, around the mycelium and ascocarp primordia, in order to favor their growth and development. The abovementioned findings make us speculate that inoculations with *P. mandelii* #29 or a mix of organic acid releaser rhizobacteria in plantations at the end of winter might improve crop yield of desert truffles.

In conclusion, the cultivable PGPR composition varied at different phenological stages of desert truffle plants. Summer was the season with the lowest microbial activity, whereas spring was the most active season. Among the PGPR traits analyzed, P-solubilizing and ACCD activity seemed to play a role in the two key annual periods (autumn and spring) of the phenological cycle of mycorrhizal plants. According to the results, applications as biofertilizers of organic acid–releasing bacteria at the end of winter could help to promote desert truffle yield.

## Supplementary Information

Below is the link to the electronic supplementary material.

Supplementary file1 (DOCX 46.5 KB)

## Data Availability

The datasets generated during and/or analyzed during the current study are available from the corresponding author on reasonable request.
