# Supplementary material for: Desert truffle mycorrhizosphere harbors organic acid releasing plant growth–promoting rhizobacteria, essentially during the truffle fruiting season
Source: Mycorrhiza. 2022 Jan 18;32(2):193–202. doi: 10.1007/s00572-021-01067-w (PMC8907101; doi:10.1007/s00572-021-01067-w)
Supplement: Supplementary file 1 — Supplementary file1 (DOCX 46.5 KB) [file 572_2021_1067_MOESM1_ESM.docx]

**Table S1**. Relative abundance of isolated bacteria by OTUs and season

| **OTU_ID** | | **Taxon** | **Isolates** | | | |  | **Percentage (%)** | **Cumulative (%)** |
| --- | --- | --- | --- | --- | --- | --- | --- | --- | --- |
|  |  |  | Autumn | Winter | Spring | Summer | Total |  |  |
| **#36** | *Pseudomonas* sp. | | 2 | 9 | 30 | - | 41 | 9.8 | 9.8 |
| **#14** | *Sinorhizobium* sp. | | 39 | - | - | - | 39 | 9.4 | 19.2 |
| **#13** | *Actinomyces* sp. | | 7 | 3 | - | 20 | 30 | 7.2 | 26.4 |
| **#61** | *Bacillus* sp. | | 13 | - | 4 | 10 | 27 | 6.5 | 32.9 |
| **#8** | *Streptomyces* sp. | | 8 | 7 | - | 10 | 25 | 6.0 | 38.8 |
| **#10** | *Streptomyces* sp. | | 8 | 6 | - | 11 | 25 | 6.0 | 44.8 |
| **#9** | *Streptomyces* sp. | | 7 | 7 | - | 10 | 24 | 5.8 | 50.6 |
| **#24** | *Variovorax* sp. | | - | 9 | 4 | 1 | 14 | 3.4 | 54.0 |
| **#43** | *Paenibacillus* sp. | | - | 2 | 10 | - | 12 | 2.9 | 56.8 |
| **#27** | *Pseudomonas* sp. | | - | 7 | 5 | - | 12 | 2.9 | 59.7 |
| **#67** | *Staphylococcus* sp. | | - | - | - | 9 | 9 | 2.2 | 61.9 |
| **#46** | *Bacillus* sp. | | - | 4 | - | 3 | 7 | 1.7 | 63.5 |
| **#48** | *Bacillus* sp. | | 1 | 4 | - | - | 5 | 1.2 | 64.7 |
| **#53** | *Bacillus* sp. | | - | - | 2 | 3 | 5 | 1.2 | 65.9 |
| **#57** | *Bacillus* sp. | | 5 | - | - | - | 5 | 1.2 | 67.1 |
| **#39** | *Paenibacillus* sp. | | - | - | 5 | - | 5 | 1.2 | 68.3 |
| **#40** | *Paenibacillus* sp. | | - | - | 5 | - | 5 | 1.2 | 69.5 |
| **#41** | *Paenibacillus* sp. | | - | - | 5 | - | 5 | 1.2 | 70.7 |
| **#33** | *Pseudomonas* sp. | | - | - | 5 | - | 5 | 1.2 | 71.9 |
| **#35** | *Pseudomonas* sp. | | - | - | 5 | - | 5 | 1.2 | 73.1 |
| **#68** | *Staphylococcus* sp. | | - | - | - | 5 | 5 | 1.2 | 74.3 |
| **#12** | *Streptomyces* sp. | | - | 3 | 2 | - | 5 | 1.2 | 75.5 |
| **#26** | *Acinetobacter* sp. | | - | 4 | - | - | 4 | 1.0 | 76.5 |
| **#31** | *Pseudomonas* sp. | | - | - | 4 | - | 4 | 1.0 | 77.5 |
| **#32** | *Pseudomonas* sp. | | - | - | 4 | - | 4 | 1.0 | 78.4 |
| **#34** | *Pseudomonas* sp. | | - | - | 4 | - | 4 | 1.0 | 79.4 |
| **#66** | *Staphylococcus* sp. | | - | - | - | 4 | 4 | 1.0 | 80.3 |
| **#3** | *Arthrobacter* sp. | | - | 3 | - | - | 3 | 0.7 | 81.1 |
| **#4** | *Arthrobacter* sp. | | - | 3 | - | - | 3 | 0.7 | 81.8 |
| **#6** | *Arthrobacter* sp. | | - | 3 | - | - | 3 | 0.7 | 82.5 |
| **#54** | *Bacillus* sp. | | - | - | 2 | 1 | 3 | 0.7 | 83.2 |
| **#55** | *Bacillus* sp. | | 3 | - | - | - | 3 | 0.7 | 83.9 |
| **#56** | *Bacillus* sp. | | 3 | - | - | - | 3 | 0.7 | 84.7 |
| **#58** | *Bacillus* sp. | | 3 | - | - | - | 3 | 0.7 | 85.4 |
| **#59** | *Bacillus* sp. | | 3 | - | - | - | 3 | 0.7 | 86.1 |
| **#60** | *Bacillus* sp. | | 3 | - | - | - | 3 | 0.7 | 86.8 |
| **#19** | *Bradyrhizobium* sp. | | 3 | - | - | - | 3 | 0.7 | 87.5 |
| **#15** | *Sinorhizobium* sp. | | 3 | - | - | - | 3 | 0.7 | 88.2 |
| **#18** | *Sinorhizobium* sp. | | 1 | 2 | - | - | 3 | 0.7 | 89.0 |
| **#20** | *Sinorhizobium* sp. | | 3 | - | - | - | 3 | 0.7 | 89.7 |
| **#65** | *Staphylococcus* sp. | | - | - | - | 3 | 3 | 0.7 | 90.4 |
| **#1** | *Arthrobacter* sp. | | - | 2 | - | - | 2 | 0.5 | 90.9 |
| **#5** | *Arthrobacter* sp. | | - | 2 | - | - | 2 | 0.5 | 91.4 |
| **#45** | *Bacillus* sp. | | - | 2 | - | - | 2 | 0.5 | 91.8 |
| **#47** | *Bacillus* sp. | | - | 2 | - | - | 2 | 0.5 | 92.3 |
| **#51** | *Bacillus* sp. | | - | - | - | 2 | 2 | 0.5 | 92.8 |
| **#44** | *Paenibacillus* sp. | | 2 | - | - | - | 2 | 0.5 | 93.3 |
| **#30** | *Pseudomonas* sp. | | - | 2 | - | - | 2 | 0.5 | 93.8 |
| **#16** | *Sinorhizobium* sp. | | 1 | 1 | - | - | 2 | 0.5 | 94.2 |
| **#17** | *Sinorhizobium* sp. | | 1 | 1 | - | - | 2 | 0.5 | 94.7 |
| **#21** | *Sinorhizobium* sp. | | 1 | 1 | - | - | 2 | 0.5 | 95.2 |
| **#22** | *Sinorhizobium* sp. | | 1 | 1 | - | - | 2 | 0.5 | 95.7 |
| **#64** | *Staphylococcus* sp. | | - | - | - | 2 | 2 | 0.5 | 96.2 |
| **#11** | *Streptomyces* sp. | | - | 1 | 1 | - | 2 | 0.5 | 96.6 |
| **#2** | *Arthrobacter* sp. | | - | 1 | - | - | 1 | 0.2 | 96.9 |
| **#49** | *Bacillus* sp. | | *-* | 1 | - | - | 1 | 0.2 | 97.1 |
| **#50** | *Bacillus* sp. | | *-* | 1 | - | - | 1 | 0.2 | 97.4 |
| **#52** | *Bacillus* sp. | | - | - | - | 1 | 1 | 0.2 | 97.6 |
| **#37** | *Brevibacillus* sp. | | - | 1 | - | - | 1 | 0.2 | 97.8 |
| **#23** | *Chitinophaga* sp. | | - | - | - | 1 | 1 | 0.2 | 98.1 |
| **#7** | *Micrococcus* sp. | | - | - | 1 | - | 1 | 0.2 | 98.3 |
| **#38** | *Paenibacillus* sp. | | 1 | - | - | - | 1 | 0.2 | 98.6 |
| **#42** | *Paenibacillus* sp. | | 1 | - | - | - | 1 | 0.2 | 98.8 |
| **#28** | *Pseudomonas* sp. | | - | 1 | - | - | 1 | 0.2 | 99.0 |
| **#29** | *Pseudomonas* sp. | | - | - | 1 | - | 1 | 0.2 | 99.3 |
| **#62** | *Staphylococcus* sp. | | *-* | 1 | - | - | 1 | 0.2 | 99.5 |
| **#63** | *Staphylococcus* sp. | | 1 | - | - | - | 1 | 0.2 | 99.8 |
| **#25** | *Stenotrophomonas* sp. | | - | 1 | - | - | 1 | 0.2 | 100.0 |

**Table S2**. Summary of the percentages (%) of bacteria during seasons of 417 cultured strains based on microscopy and biochemical phenotype characterization. ND: not detectable.

|  |  | **% Gram-positive** | | | |  | **% Gram-negative** | | |
| --- | --- | --- | --- | --- | --- | --- | --- | --- | --- |
|  | **CFU g^-1^** | Actinobacteria | Spore-forming rods | Cocci | Total |  | Oxidase negative | Oxidase positive | Total |
| **Total** |  | 30.2 | 26 | 0.8 | 57 |  | 24 | 19 | 43 |
| **Summer** | 1.61 ± 0.6 x10^6^ | 53 | 21 | 1 | 75 |  | 25 | ND | 25 |
| **Autumn** | 4.52 ± 3.0 x10^7^ | 24 | 31 | 1 | 56 |  | 42 | 2 | 44 |
| **Winter** | 3.28 ± 1.3 x10^6^ | 41 | 17 | 1 | 59 |  | 21 | 20 | 41 |
| **Spring** | 2.03 ± 1.5 x10^6^ | 3 | 34 | 1 | 38 |  | 4 | 58 | 62 |

**Table S3.** Characterization of plant growth-promoting traits in the 68 different OTUs generated from bacterial colonies for auxin production (IAA), phosphate solubilization (P solubilization), siderophore production or ACC deaminase production (ACCD). (-) PGRP trait not detected, (+) PGPR trait detected.

| OTU_ID | Taxon | IAA | P solubilization | Siderophore | ACCD |
| --- | --- | --- | --- | --- | --- |
| #01 | *Arthrobacter* sp. | - | - | - | - |
| #02 | *Arthrobacter* sp. | - | - | + | - |
| #03 | *Arthrobacter* sp. | + | - | - | + |
| #04 | *Arthrobacter* sp. | + | - | - | + |
| #05 | *Arthrobacter* sp. | + | - | - | - |
| #06 | *Arthrobacter* sp. | + | - | - | + |
| #07 | *Micrococcus* sp. | + | - | + | + |
| #08 | *Streptomyces* sp. | - | - | - | - |
| #09 | *Streptomyces* sp. | - | - | - | - |
| #10 | *Streptomyces* sp. | - | - | - | - |
| #11 | *Streptomyces* sp. | - | - | - | - |
| #12 | *Streptomyces* sp. | - | - | + | - |
| #13 | *Actinomyces* sp. | - | - | - | - |
| #14 | *Sinorhizobium* sp. | - | - | - | - |
| #15 | *Sinorhizobium* sp. | - | - | + | + |
| #16 | *Sinorhizobium* sp. | - | - | + | - |
| #17 | *Sinorhizobium* sp. | - | - | + | - |
| #18 | *Sinorhizobium* sp. | - | - | - | - |
| #19 | *Bradyrhizobium* sp. | - | - | - | - |
| #20 | *Sinorhizobium* sp. | - | - | - | - |
| #21 | *Sinorhizobium* sp. | - | - | + | - |
| #22 | *Sinorhizobium* sp. | - | - | - | - |
| #23 | *Chitinophaga* sp. | - | - | - | - |
| #24 | *Variovorax* sp. | - | - | - | - |
| #25 | *Stenotrophomonas* sp. | - | - | + | + |
| #26 | *Acinetobacter* sp. | - | + | - | - |
| #27 | *Pseudomonas* sp. | - | + | - | + |
| #28 | *Pseudomonas* sp. | - | + | + | + |
| #29 | *Pseudomonas* sp. | - | + | - | - |
| #30 | *Pseudomonas* sp. | - | + | - | + |
| #31 | *Pseudomonas* sp. | + | + | - | - |
| #32 | *Pseudomonas* sp. | - | + | - | - |
| #33 | *Pseudomonas* sp. | - | + | + | + |
| #34 | *Pseudomonas* sp. | + | + | - | - |
| #35 | *Pseudomonas* sp. | + | + | - | - |
| #36 | *Pseudomonas* sp. | - | + | - | + |
| #37 | *Brevibacillus* sp. | + | - | + | - |
| #38 | *Paenibacillus* sp. | - | - | - | - |
| #39 | *Paenibacillus* sp. | - | - | - | - |
| #40 | *Paenibacillus* sp. | - | - | - | - |
| #41 | *Paenibacillus* sp. | - | - | - | - |
| #42 | *Paenibacillus* sp. | - | - | - | + |
| #43 | *Paenibacillus* sp. | - | - | - | - |
| #44 | *Paenibacillus* sp. | - | - | - | + |
| #45 | *Bacillus* sp. | - | - | - | + |
| #46 | *Bacillus* sp. | - | - | - | - |
| #47 | *Bacillus* sp. | - | - | + | + |
| #48 | *Bacillus* sp. | - | - | - | - |
| #49 | *Bacillus* sp. | - | + | - | - |
| #50 | *Bacillus* sp. | - | + | - | - |
| #51 | *Bacillus* sp. | - | - | + | + |
| #52 | *Bacillus* sp. | + | + | - | + |
| #53 | *Bacillus* sp. | - | + | - | + |
| #54 | *Bacillus* sp. | - | + | - | + |
| #55 | *Bacillus* sp. | - | - | - | - |
| #56 | *Bacillus* sp. | - | - | + | - |
| #57 | *Bacillus* sp. | + | - | - | - |
| #58 | *Bacillus* sp. | - | - | + | - |
| #59 | *Bacillus* sp. | - | - | + | + |
| #60 | *Bacillus* sp. | - | - | - | - |
| #61 | *Bacillus* sp. | - | - | - | - |
| #62 | *Staphylococcus* sp. | - | - | - | - |
| #63 | *Staphylococcus* sp. | - | - | - | + |
| #64 | *Sinorhizobium* sp. | - | - | - | - |
| #65 | *Staphylococcus* sp. | - | - | - | - |
| #66 | *Staphylococcus* sp. | - | - | - | - |
| #67 | *Staphylococcus* sp. | - | - | - | - |
| #68 | *Staphylococcus* sp. | - | - | + | - |

**Table S4**. Relative abundance of isolated bacteria by genus and season.

| **Genus** | **Isolates** | | | | | **Percentage**  **(%)** | **Cumulative (%)** |
| --- | --- | --- | --- | --- | --- | --- | --- |
|  | Autumn | Winter | Spring | Summer | Total |  |  |
| *Streptomyces* | 23 | 24 | 3 | 31 | 81 | 19.4 | 19.4 |
| *Pseudomonas* | 2 | 19 | 58 | - | 79 | 18.9 | 38.4 |
| *Bacillus* | 34 | 14 | 8 | 20 | 76 | 18.2 | 56.6 |
| *Sinorhizobium* | 50 | 6 | - | - | 56 | 13.4 | 70.0 |
| *Paenibacillus* | 4 | 2 | 25 | - | 31 | 7.4 | 77.5 |
| *Actinomyces* | 7 | 3 | - | 20 | 30 | 7.2 | 84.7 |
| *Staphylococcus* | 1 | 1 | - | 23 | 25 | 6.0 | 90.6 |
| *Arthrobacter* | - | 14 | - | - | 14 | 3.4 | 94.0 |
| *Variovorax* | - | 9 | 4 | 1 | 14 | 3.4 | 97.4 |
| *Acinetobacter* | - | 4 | - | - | 4 | 1.0 | 98.3 |
| *Bradyrhizobium* | 3 | - | - | - | 3 | 0.7 | 99.0 |
| *Brevibacillus* | - | 1 | - | - | 1 | 0.2 | 99.3 |
| *Chitinophaga* | - | - | - | 1 | 1 | 0.2 | 99.5 |
| *Micrococcus* | - | - | 1 | - | 1 | 0.2 | 99.8 |
| *Stenotrophomonas* | - | 1 | - | - | 1 | 0.2 | 100.0 |

**Table S5.1**. Permutational analysis of variance (PERMANOVA) testing the effect of sampling time (i.e. season) on bacterial community composition.

|  | **Df** | **Sums of Sqs** | **Mean Sqs** | **F. Model** | **R^2^** | **Pr(>F)** |
| --- | --- | --- | --- | --- | --- | --- |
| **Season** | 3 | 4.084 | 1.361 | 2.706 | 0.474 | 0.001 |
| **Residuals** | 9 | 4.528 | 0.503 |  | 0.526 |  |
| **Total** | 12 | 8.613 |  |  | 1.000 |  |

**Table S5.2**. Permutational test for RLQ model, testing the significance of the relationship between the OTU abundance, trait and environmental matrices. Null model #2 permuted values of sites and null model #4 permuted values of species in the OTU abundance table.

|  | **Observation** | **St. Obs.** | **Alternative model** | **P value** |
| --- | --- | --- | --- | --- |
| **Model #2** | 0.514 | 3.034 | Two-sided | 0.0045 |
| **Model #4** | 0.514 | 4.698 | Two-sided | 0.0004 |
